# Quantum versus simulated annealing in wireless interference network optimization

**DOI:** 10.1038/srep25797

**Published:** 2016-05-16

**Authors:** Chi Wang, Huo Chen, Edmond Jonckheere

**Affiliations:** 1 Department of Electrical Engineering, University of Southern California, Los Angeles, CA90089, USA.

## Abstract

Quantum annealing (QA) serves as a specialized optimizer that is able to solve many NP-hard problems and that is believed to have a theoretical advantage over simulated annealing (SA) via quantum tunneling. With the introduction of the D-Wave programmable quantum annealer, a considerable amount of effort has been devoted to detect and quantify quantum speedup. While the debate over speedup remains inconclusive as of now, instead of attempting to show general quantum advantage, here, we focus on a novel real-world application of D-Wave in wireless networking—more specifically, the scheduling of the activation of the air-links for maximum throughput subject to interference avoidance near network nodes. In addition, D-Wave implementation is made error insensitive by a novel Hamiltonian extra penalty weight adjustment that enlarges the gap and substantially reduces the occurrence of interference violations resulting from inevitable spin bias and coupling errors. The major result of this paper is that quantum annealing benefits more than simulated annealing from this gap expansion process, both in terms of ST99 speedup and network queue occupancy. It is the hope that this could become a real-word application niche where potential benefits of quantum annealing could be objectively assessed.

## Quantum Annealing

Quantum annealing is designed to mimic the process of simulated annealing^1^ as a generic way to efficiently get close-to-optimum solutions in many NP-hard optimization problems. Quantum annealing is believed to utilize quantum tunneling instead of thermal hopping to more efficiently search for the optimum solution in the Hilbert space of a quantum annealing device such as the D-Wave^2^,^3^. Since the introduction of D-Wave One in 2011, there has been a considerable amount of work done on its potential applications, e.g., protein folding^4^, determining Ramsey number^5^, Bayesian network structure learning^6^, operational planning^7^, power system fault detection^8^, graph isomorphism^9^, database optimization^10^, machine learning^11^, deep learning^12^, to name but a few. Note that of all the applications we are aware of, most were solved with small logical instance size due to hardware constraints and none of them demonstrated conclusive speedup compared to classical methods. In fact, benchmarking quantum speedup is more subtle than it appears to be. There has recently been a lot of insightful work on such a challenging question^13^,^14^,^15^. One conclusive remark is that no evidence for a general speedup has been found thus far, but potential speedup might exist in certain problems or upon scaling. In this paper, we introduce a new breed of graph-theoretic applications motivated by the very problem that makes the design of optimal wireless network protocols outstanding from the computational viewpoint—the interference constraints—and we show that a gap expansion technique benefits quantum annealing much more than simulated annealing, indicating a way to obtain quantum speedup.

Quantum annealing is based on the premise that the minimum energy configuration of the Ising spin glass model encodes the solution to specific NP-hard problems, including all Karp’s 21 NP-complete problems^16^. Such problems are written in the form of Quadratic Unconstrained Binary Optimization (QUBO) problems,


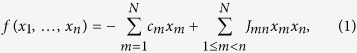


where *x*_*m*_ ∈ {0, 1}, and *c*_*m*_, *J*_*mn*_ represent QUBO parameters. The latter has an equivalent Ising formulation


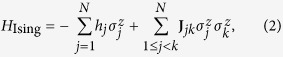


where 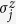 represents the *z*-Pauli operator of Ising spin *j* in the network, and *h*_*j*_ and **J**_*jk*_ are local fields and coupling strengths, resp. The annealer initially prepares a initial transverse magnetic field, an equal superposition of 2^*N*^ computational basis states, eigenspace of


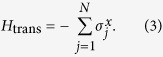


During the adiabatic continuation, the Hamiltonian evolves smoothly from *H*_trans_ to *H*_Ising_, that is,


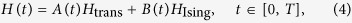


where *A*(·) decreases monotonically from 1 to 0 and *B*(·) increases monotonically from 0 to 1. From the adiabatic theorem, if the evolution is “slow enough”, the system would remain in its ground state. Thus, in principle, an optimal solution to the original problem could be obtained by measurements at final time^17^.

## Network Scheduling Problem

One of the fundamental problems in multi-hop wireless networks is network scheduling. Signal-to-Interference-plus-Noise Ratio (SINR) has to be maintained above a certain threshold to ensure successful decoding of information at the destination. For example, IEEE 802.11b requires a minimum SINR of 4 and 10 dB corresponding to 11 and 1 Mbps channel, resp^18^. Consequently, only a subset of edges of the network can be activated within the same time slot, since every link transmission causes interference with nearby link transmissions.

Different networks and protocols use different interference models. The most commonly used model is the 1-hop interference model (node exclusive model), in which every node can transmit or receive along only one activated link abutting that node in the same time slot. This is commonly used in Bluetooth and FH-CDMA^19^,^20^, while the 2-hop interference model is sometimes used in studies of IEEE 802.11 networks^21^, in which no two links within 2-hop distance can be simultaneously activated. Generally, *K*-hop is used with *K* representing arbitrary hop interference model. In some cross-layer optimization studies^22^, the optimal *K* is found to vary between 1 and 3. In theory, protocol designers can set *K* to arbitrary value, as long as end-to-end delay, signal interference and computation cost reach a Pareto-optimal point.

There are two major methods for solving network interference: the first one is the graph based model that solves the Weighted Maximum Independence Set (WMIS) problem on a conflict graph^23^,^24^,^25^,^26^,^27^, the other optimizes a geometric-based SINR^28^,^29^,^30^,^31^. The former is sometimes argued as being an overly idealistic assumption; however, the WMIS problem is still involved in the latter model^32^ and is of interest in SA versus QA; thus we mainly consider the former model for simplicity. Several general heuristics for the WMIS problem are well-studied, including simulated annealing^33^, neural networks^34^,^35^,^36^, genetic algorithm^37^,^38^,^39^, greedy randomized genetic search^40^, Tabu search^41^,^42^,^43^,^44^. It is claimed that simulated annealing is superior to other competing methods with experimental instances of up to 70,000 nodes^33^.

To give formal definitions on a graph *G* = (*V*, *E*), let *w*_*l*∈*E*_ be the wireless networking link weights in a given time slot. (They are related to queue differential and somehow the most highly weighted links should receive priority activation if interference allows.) Let *d*_*S*_(*x*, *y*) denote the hop distance between *x*, *y* ∈ *V*. Consider edges *e*_*u*_, *e*_*v*_ ∈ *E* and let ∂*e*_*u*_ = {*u*_1_, *u*_2_}, ∂*e*_*v*_ = {*v*_1_, *v*_2_}. We then define


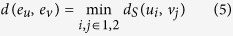


to be the distance between edges. Similar to the definition in the work of Sharma *et al*.^22^, a subset of edges *E*′ is said to be valid subject to the *K*-hop interference model if, for all *e*_1_, *e*_2_ ∈ *E*′ with *e*_1_ ≠ *e*_2_, we have *d*(*e*_1_, *e*_2_) ≥ *K*. Let *S*_*K*_ denote the set of *K*-hop valid edge subsets (scheduling set). Then the network scheduling under the *K*-hop interference model is


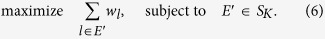


In the *K* = 1 case, the problem is a max-weight matching problem and thus has polynomial solution (Edmonds’ blossom algorithm^45^). However, for the case *K* > 1, the problem is proved to be NP-hard and non-approximable^22^. As already said, the network scheduling problem (6) has to be solved in every time slot; thus, the complexity of the exact scheduling problem becomes critical in time-sensitive applications.

## Overview

We intend to design a quantum annealing scheduler to solve the WMIS problem and benchmark the results obtained by realistic network simulation against simulated annealing. The complete benchmarking is summarized in the following steps.

### Conflict Graph

**Figure 1:**
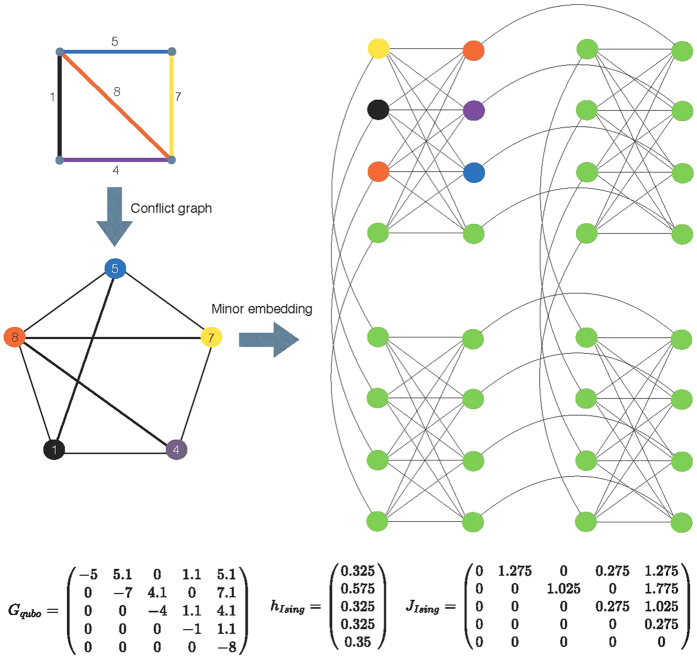
An example of conflict graph conversion, QUBO generation, and minor-embedding to Chimera architecture. The conflict graph is generated based on the 1-hop interference model. Only 4 cells of *K*_4,4_ structure of the Chimera architecture are shown. On D-Wave Two platform, 64 such cells are available, giving 512 available physical qubits. Quantum annealing is performed on such architecture to theoretically give the ground state of the configuration, and thus the network scheduling solution to the original problem. The numerical data shows the conflict graph weight structure *G*_qubo_ and how it is mapped to the Ising Hamiltonian characterized by *h*_Ising_ and **J**_Ising_. The off-diagonal entries of *G*_qubo_ are chosen as 0.1 larger than the minimum of the weights of the two corresponding vertices of the conflict graph.

The original scheduling problem on the original graph is reformulated as a QUBO problem (1) on a conflict graph: the nodes of the conflict graph are the edges of the original graph with weights *w*_*l*_ (=*c*_*l*_ in QUBO), while the edges of the conflict graph are pairs of edges of the original graph in scheduling conflict, as illustrated in Fig. 1, left. As such, subject to proper choice of the *J*_*mn*_ (details in supplemental material), the QUBO problem (1) on the conflict graph, the WMIS problem, provides a solution to *K*-hop interference problem on the original graph (6).

### Gap Expansion

In the WMIS problem, some *β*_*mn*_ scaling of *J*_*mn*_ is introduced as shown in (7) to open up the gap of the Ising model, resulting in less scheduling violations, especially in the QA approach.

### Embedding to Hardware

The QUBO problem has to be mapped to the Ising Hamiltonian via 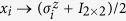, with the constant energy shift ignored. We perform minor embedding into the D-Wave Chimera architecture, as illustrated in Fig. 1, right, or decide that it is not embeddable. This is discussed in more detail in the supplemental material. Figure 1 gives a simple numerical example of how a network is mapped to D-Wave architecture.

### Measurement and Post-processing

We perform the annealing run a certain number of times on D-Wave, find the lowest energy configuration, and use such configuration as scheduling solution. Some commonly used algorithm techniques in adiabatic quantum computation, such as gauge transformation and majority vote on possible broken chains are not performed. See the work of King *et al*.^46^ for more discussion.

## Results

In theory and practice, SA is regarded as a strong competitor of QA and their performances are often contrasted in the literature^13^,^14^,^15^. On the other hand, it is known that SA can also boost the overall performance of classical heuristic algorithms in solving WMIS problems^33^. Thus, it is worth comparing our QA results with the performance of the SA algorithm. Comparison metrics are defined later in this section. We use the gap expansion process on the quantum scheduler, defined in detail in the following section, and observe that the quantum scheduler takes better advantage of this gap expansion technique than SA.

Table 1 shows the parameters of the 2 random graphs (15 and 20 nodes) being tested on D-Wave installed at Information Sciences Institute of University of Southern California (USC). The “problem size” *n* is the number of nodes of the wireless network, the “QUBO size” is |*E*| and thus generally scales as *O*(*n*^2^), where *E* and *n* are the edge set and order of the problem graph, resp. The “Physical Qubits” is the number of nodes *N* after minor embedding in the D-Wave architecture. After minor-embedding in the case of the 20-node network, a significantly large proportion of all available qubits are utilized (405 out of 502).

### Simulated Annealing Setup

Here, we adapted a highly optimized simulated annealing algorithm (an_ss_ge_fi_vdeg)^47^, compiling the C++ source code with gcc 4.8.4 with MATLAB C-mex API. Additionally, a wide range of parameters were tested to ensure near optimal performance of the algorithm within a reasonable run time (see supplemental material). All the non-time-critical tests in this section were ran on the servers of the USC Center for High-Performance Computing (HPC).

### Quality metrics

We compare the performance of QA and SA based on three quantitative metrics: average network delay, throughput optimality, and ST99. The first metric reflects the actual network performance; the latter two metrics reflect the accuracy and speed of QA and SA compared to exact solvers (see supplemental material for formal definitions.)

#### Average network delay

**Figure 2:**
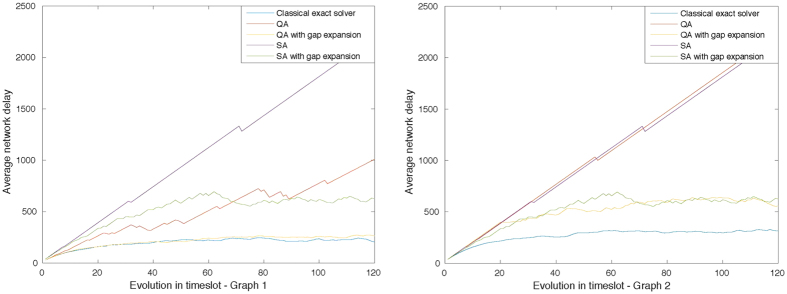
Network delay of classical exact algorithm, quantum annealing, quantum annealing after gap expansion, and simulated annealing are plotted versus time slot for two randomly generated networks. The left one is a simpler case with 15 nodes and 31 edges where quantum annealing reaches almost as good a result as the exact solution in terms of network delay; the right one is a harder case with 20 nodes and 57 edges in total.

We use an optimized wireless network protocol in our experiment (see supplemental material for protocol definitions). In Fig. 2, we show that, after gap expansion, the overall performance significantly improves. As one would expect, the network delay is also improved and gets closer to the exact classical solution. It is a rather delicate issue as how to deal with non-independent solutions returned by D-Wave. In the experiment, we skip the time slot transmission if the lowest energy solution does not conform to the *K*-hop interference model. Sometimes, it makes sense to perform a polynomial greedy style matching when the returned result is non-independent, in which case the problem is more error-insensitive and thus more scalable. Here we consider the worst case scenario, that is, no transmission is allowed if the result is non-independent.

#### Throughput optimality and ST99[OPT]

**Figure 3:**
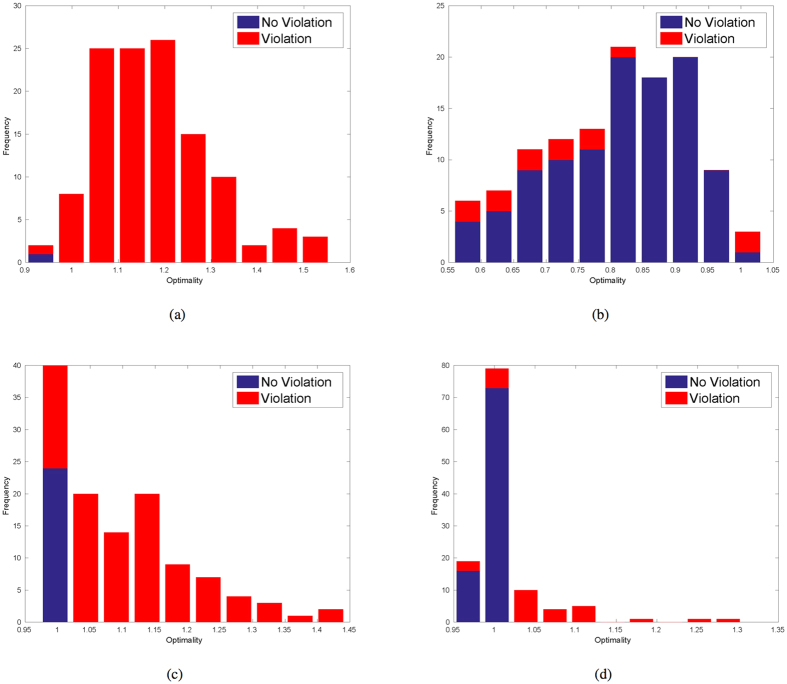
Number of violation (red) and nonviolation (blue) cases versus throughput optimality measure on test graph 2 (the harder instance). D-Wave simulation runs are shown in (**a**,**b**). SA simulation runs shown in (**c**,**d**) are executed under the one set of optimal parameters we have tested (5000 sweeps, 5000 repetitions, linear schedule with initial temperature *β*_0_ = 0.1 and final temperature *β*_1_ = 10). Note that the abscissa of the blue vertical bars register *F*_quantum_ whereas the abscissa of the red vertical bars registers 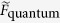 as defined in supplemental material. (**a**) D-Wave: Before gap expansion; (**b**) D-wave: After gap expansion; (**c**) Simulated Annealing: Before gap expansion; (**d**) Simulated Annealing: After gap expansion.

Based on our previous assumption, it is of vital importance that the solutions given by the solver satisfy the independence constraint. Here we use the optimality measure *F*_quantum_ as defined in section 5.2 of supplemental material. Briefly, if there are no violations, such measure reaches 1 if the solution is exactly optimal, and less than 1 if suboptimal. If there are violations, 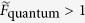 if those solutions improve the throughput and 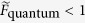 if the violations do not even improve the throughput. In Fig. 3, we investigate the distribution of the optimality factors among all 120 time slots of Graph 2 (the harder graph). All the violations are counted inside red bars. Comparing (a) and (c) of Fig. 3 and (b) with (d) of Fig. 3, we can see that energy compression as shown in Fig. 2 results in substantially less violations of the independence constraint, and that this reduction is more pronounced in the QA case than in the SA case. This could explain the huge performance improvement before and after gap expansion in Fig. 2. The test results of SA under a particular set of parameters are shown in (c) and (d) of Fig. 3 The ranges of our tested parameters are listed in the supplemental material. It is worth making the following observations:

1. *QA benefits more from the gap expansion compared to SA*.
2. *SA either finds the very optimal ground state or gets trapped in very ‘deep’ local minima*.

There is still a relatively large number of violations in the returned results of SA even after gap expansion. This is in agreement with our previous knowledge about SA in that it is designed to find the ground state and it is not optimized to search for sub-optimal results. In addition, Table 2 shows the exact numbers related to the performances of SA and QA after gap expansion.

**Figure 4:**
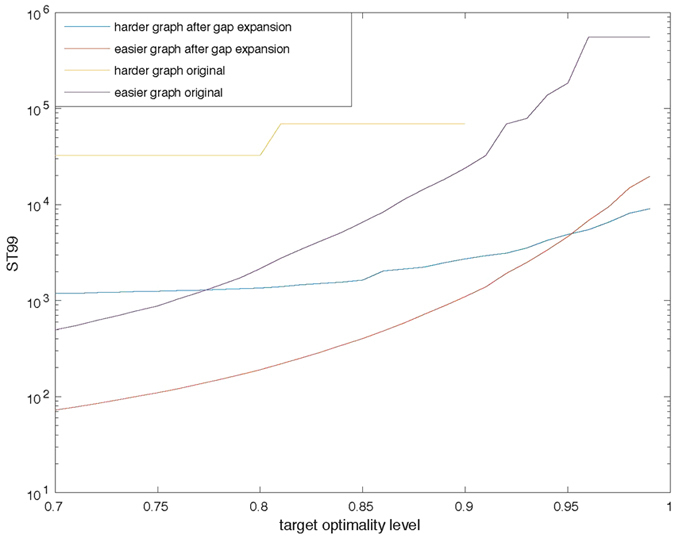
ST99, defined as 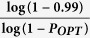, versus required optimality level OPT specified by network, before and after gap expansion on Graph 1 and Graph 2 (the harder instance). Note that the probability is calculated based on all solutions returned by D-Wave; thus, an ST99 of 10^3^ corresponds to one set of annealing runs, which in our case costs 20 ms (1000 annealing runs with 20 *μ*s for each run). Curve for Graph 2 before gap expansion ends at 0.9 optimality level, because there are no solutions that satisfy such optimality level after a total of 120,000 annealing runs.

From the optimality data returned by D-Wave, we can plot ST99 related to the level of optimality as shown in Fig. 4. Note that our ST99 definition (see supplemental material) relies on optimality level, which could typically be 80% or 90% depending on user needs.

In Table 3, we show how setting the penalty weight *β* of the gap expansion consistently with the local or global approach would affect the quality of the returned solution. We found that setting *β*_global_ = 1 would yield the best performance so far. We do not have a quantitative explanation for the wrong solutions; potential explanations on small problems have been discussed in refs 13, 14, 15. Intuitively, as the problem size grows, it is much more difficult even to find close to ground states; indeed, as the penalty weight grows too large, the local fields begin to vanish, thus making the problem effectively more difficult since all weights have to be scaled to [−2, +2]. The problem of quantitative connection among quality measure, network stability, and throughput optimality remains open.

## Methods

### Gap Expansion

**Figure 5:**
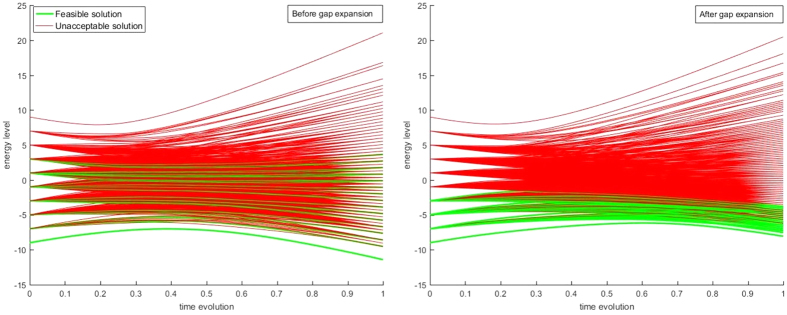
Hamiltonian energy level evolution with *H*(*s*) = (1 − *s*)*H*_trans_ + *sH*_Ising_, demonstrating the feasibility of energy compression on a small, randomly generated test wireless graph with 7 nodes on the conflict graph, plotting all 128 possible energy states in both before and after gap expansion cases. Both Hamiltonians are rescaled to have a maximum energy level of 2 for both coupling strengths and local fields. Note that for a typical desktop computer, such plots are impossible to construct once the size of the conflict graph goes higher than 12.

We try to make the problem insensitive to quantum annealing errors by properly setting the *h*_*i*_ and **J**_*ij*_ terms in Eq. (2) in order to expand the **final** energy gap. Note that this gap is the energy gap between the eigenvalues of the Ising form at the **end** of the adiabatic evolution, not to be confused with the gap between ground and first excited states **during** the evolution. We introduce a scaling factor *β*_*mn*_ that multiplies the quadratic part of the QUBO formulation and as such scales the various terms so as to put more penalty weight on the independence constraints:


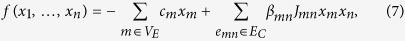


where *V*_*E*_ and *E*_*C*_ denote the vertex set and edge set, resp., of the conflict graph and *β*_*mn*_ is chosen uniform. In theory, *β*_*mn*_ = 1 would suffice as long as *J*_*mn*_ > min(*c*_*m*_, *c*_*n*_), as the ground state has already encoded the correct solution of the WMIS problem^48^. However, since measuring the ground state correctly is not guaranteed, increasing *β*_*mn*_ becomes necessary to enforce the independence constraints, so that the energy spectrum of the non-independence states is raised to the upper energy spectrum and the feasible energy states are compressed to the lower spectrum. Figure 5 illustrates the effect of the feasible energy compression on a small random graph.

A natural question regarding *β* would be, How large is large enough? The problem is, interestingly, another optimization problem itself. The D-Wave V7 is subject to an Internal Control Error (ICE) that gives Gaussian errors with standard deviations *σ*_*h*_ ≈ 0.05 and *σ*_**J**_ ≈ 0.035^46^. Putting too large a *β*_*mn*_ penalty would incur two problems: 1) The local fields would become indistinguishable and 2) The minimum evolution gap would become too small. Accordingly, a few parameter values have been tried out and the results are shown in Table 3, which corroborates the experimental results of the previous section indicating that gap expansion would significantly influence the optimality of the returned solution.

There exist several strategies in setting heavier penalty weights to expand the gap. We compare two different approaches to set the penalty weights:


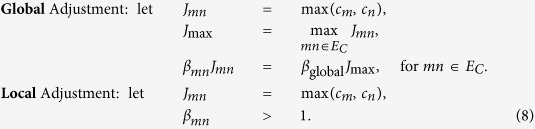


In the local adjustment^48^, the constraint on *J*_*mn*_ depends only on the fields at ∂*mn*, whereas in the global adjstment, contrary to^48^, *J*_*mn*_ depends on *all* fields.

## Discussion

We have presented the first experimental D-Wave application of network scheduling—a problem widely investigated in the field of wireless networking understood in the classical sense. The problem is reduced to a WMIS problem, itself reformulated in the form of the minimum energy level of an Ising Hamiltonian, itself mapped to the Chimera architecture by utilizing a differential geometrical approach^49^ that immediately rules out cases where minor-embedding is impossible. We increased the success rate of the quantum annealer and the simulated annealer to return non-violation solutions by classically increasing the energy gap, so that the range of acceptable solutions is greatly increased.

By comparing QA with SA, though omitting Quantum Monte Carlo (QMC), we found a potential comparison point where QA outperforms SA in the sense of benefit from gap expansion as seen in Fig. 3. Gap expansion reduces by 89% the number of violation cases for QA, while it only reduces by 67.5% the number of violation cases for SA. Although, as seen from Table 2, SA has an optimality advantage in non-violation cases, it is however interesting to observe that among suboptimal solution cases QA has less violations than SA. By deliberately constructing cases suitable for suboptimal solutions, one could potentially demonstrate a quantum advantage in terms of less violations.

As far as speed is concerned, we noted that on Graph 2, and disregarding all overhead, the 1,000 runs of QA took a total of 1000 × 20 *μ*sec = 20 msec on D-Wave II to render the solution utilized in the performance analysis. On SA on the other hand, 5,000 runs, totaling 1 minute in the adopted simulation environment, were needed to get solutions comparable with those of QA. This comparison might however be misleading as there is room for considerable speed up of SA on specialized architecture. It is also argued^14^,^15^ that even 20 *μ*s might be too large for particular problems; however, this is the minimum programmable annealing time on D-Wave platform.

Despite encouraging results, due to the very limited experimental data, we cannot positively assert a general sizable advantage of quantum annealing against simulated annealing. However, we plan to generalize this potential advantage in the future by utilizing a broader class of graphs and the 1152-qubit chip. It is hoped that this could help resolve the speedup issue^13^,^14^,^15^.

The classical wireless protocols (Backpressure, Dirichlet and Heat Diffusion, see supplemental material for protocol definition) return solutions with the interference constraints *always* satisfied—so that there is forwarding at every time slot. Here, the QA and SA interference problem solvers allow solutions that violate the interference constraints in the interest of speed. Unfortunately, should an interference constraint be violated even at a remote corner of the network, our simulation stops the transmission, so that the Dirichlet protocol as it now stands cannot take advantage of the proposed algorithm speedup. As to whether the Dirichlet protocol could be tweaked to take advantage of the faster QA solution to the interference problem is widely open.

## Additional Information

**How to cite this article**: Wang, C. *et al*. Quantum versus simulated annealing in wireless interference network optimization. *Sci. Rep*. **6**, 25797; doi: 10.1038/srep25797 (2016).

## Supplementary Material

Supplementary Information

## Figures and Tables

**Table 1:** Problem size of random graphs used in experiment.

	Problem Size	QUBO Size	Physical Qubits
Graph1	15	31	164
Graph2	20	57	405

The third column represents the actual physical qubits used after minor embedding.

**Table 2:** Performances After Gap Expansion.

	Number of violations (out of 120)	Average optimality (without violations)
Quantum Annealing	13	0.8129
Simulated Annealing	31	0.9935

**Table 3:** Penalty weight with different *β* setup and resulting quality measure of returned solution averaged over 120 time slots.

	Avg. Optimality - G1	Delay - G1	Avg. Optimality - G2	Delay - G2
*β*_*mn*_ = 1 + *ε*	0.781	384.9	0.077	NC
*β*_*mn*_ = 2	0.928	376.3	0.312	1213
*β*_global_ = 1	0.974	268	0.741	566.1
*β*_global_ = 1.5	−0.165	NC	−0.331	NC
*β*_global_ = 2	−0.185	NC	−0.356	NC

Graph 2 is a larger size instance than Graph 1. Delay refers to average network delay in steady state, and NC refers to the non-convergent case where steady state is not reached within tested time span.
