# Supplementary material for: Quantum versus simulated annealing in wireless interference network optimization
Source: Sci Rep. 2016 May 16;6:25797. doi: 10.1038/srep25797 (PMC4867427; doi:10.1038/srep25797)
Supplement: Supplementary Information [file srep25797-s1.pdf]

# SUPPLEMENTAL MATERIAL: QUANTUM VERSUS SIMULATED ANNEALING IN WIRELESS INTERFERENCE NETWORK OPTIMIZATION

CHI WANG , HUO CHEN , AND EDMOND JONCKHEERE*

**1. Wireless Networking Protocol.** Let the original wireless network graph be $G = (V, E)$, with vertices corresponding to actual physical nodes in the wireless network (such as routers or sensors) and edges being their possible physical connections. Each node receives packets in a Poisson random fashion (uniform arrival is implemented in experiments). Packets are classified according to their destination $d \in D$, where $D \subseteq V$ is a subset of sink nodes. The $d$-packet backlog in node $i$ at time $k$ will be denoted as $q_i^{(d)}(k)$ and $q_{ij}^{(d)}(k) := q_i^{(d)}(k) - q_j^{(d)}(k)$ will denote the queue backlog differential along link $ij \in E$.

We use the Dirichlet [1] protocol in the experiment in the main text, which is a variation of well-known Backpressure [13] protocol in wireless networks.

Many multi-hop wireless protocols operate on the same weighting, scheduling, forwarding strategy. The weighting assigns a weight to every link, originally meant to be the queue differential $q_{ij}^{(d)}(k)$, since links with the highest queue differentials at time slot $n$ should be among those that move packets at time slot $n$. The scheduling finds a combination of links that maximizes an aggregated weight subject to the interference constraints. The forwarding phase sends packets along those links that are activated by the scheduling process. The experimental simulation of the network scheduling based on quantum annealing can be roughly divided into the following steps:

1. **Weighting (Classical)**: For each link $ij \in E$ and destination $d$, assign a weight $w_{ij}^{(d)}(k)$ to the link. Edge weight can be defined arbitrarily as long as it incorporates the intent of the protocol. Here, the weighting follows the Dirichlet principle [1] as in Equations (2.1)-(2.2). The weighting gives a weighted undirected graph at the end of this step.
2. **Preprocessing for Scheduling (Classical)**: Convert the edge weighted graph to the conflict graph and generate the QUBO problem according to Equation (3.1). This conversion has polynomial time-complexity. However, the minor-embedding heuristics could take as long as more than 10 seconds.
3. **Scheduling by D-Wave (Quantum)**: The problem is submitted to the D-Wave at USC ISI. Each annealing run takes $20\mu$s. For each scheduling instance, 1000 annealing runs are performed, and the lowest possible energy configuration is selected.
4. **Forwarding (Classical)**: On each activated link $ij \in E'$, an amount $\widehat{f_{ij}^{(d)}}(k)$ of packets are forwarded, as in Equation (2.2).

All four steps are done within one time slot of the simulation run. The size of the time slot is typically in the scale of milliseconds in practice. It would take thousands of

*Department of Electrical Engineering, University of Southern California, Los Angeles, CA 90089.

time slots to reach steady-state under uniform packet arrival rate in a small network comprising no more than 100 nodes. Thus, one might naturally expect that, if an error occurs during one time slot in quantum annealing due to errors from either decoherence or calibration, that error would accumulate over time. However, this is not the case, as will be shown in Section 2 of the main text.

**2. Weighting.** The particular definition of edge weight makes the whole difference among many wireless network protocols. We set up our simulation based on the multi-class Dirichlet wireless protocol [1], following similar network setup [2]. The edge weight for the $d$-class (destination $d \in D$) packets is defined as

$$w_{ij}^{(d)}(k) := 2\rho_{ij}^{(d)}(k)^{-1} q_{ij}^{(d)}(k) \widehat{f_{ij}^{(d)}}(k) - \left(\widehat{f_{ij}^{(d)}}(k)\right)^2, \tag{2.1}$$

where $\rho_{ij}^{(d)}(k)$ is a link cost factor, and $\widehat{f_{ij}^{(d)}}(k)$ is defined as

$$\begin{aligned} \widehat{f_{ij}^{(d)}}(k) = \arg\min \left(\rho_{ij}^{(d)}(k)^{-1} q_{ij}^{(d)}(k) - \widehat{f_{ij}^{(d)}}(k)\right)^2 \\ \text{subject to} \\ \sum_{d \in D} \widehat{f_{ij}^{(d)}}(k) \leq \mu_{ij}(k), \quad 0 \leq \widehat{f_{ij}^{(d)}}(k) \leq q_{ij}^{(d)}(k), \end{aligned} \tag{2.2}$$

where $\mu_{ij}(k)$ is the capacity of the link $ij \in E$ at time slot $k$. $\widehat{f_{ij}^{(d)}}(k)$ is to be interpreted as the intended forwarded amount of packets on link $ij$, only actually forwarded if the interference constraints allow. It is proved that such protocol, with exact scheduling solution, is throughput optimal [1]. It is worth noting that such weighting completely depends on the network running status (real-time queue backlog) and traffic arrival rate. The latter time-varying aspect compounded with the confinement of the interference computational solution within one time slot is one of the biggest challenges to cope with in this real-world application of quantum annealing.

**3. Mapping to QUBO.** Given $G = (V, E)$, the corresponding conflict graph $G_C = (V_E, E_C)$ has its vertex set equal to the edge set of $G$ and its edge set consisting of those pairs of edges $(e_u, e_v)$ such that the hop distance between any two end vertices is $\leq K$.

To connect with the QUBO problem, a vertex of the conflict graph will be denoted as $m \in V_E$. If the vertex $m \in V_E$ in $G_C$ has end vertices $i, j \in V$ as an edge in $G$, it will be rewritten $m_{ij} \in E$ in the original graph $G$. Thus $m$ is a vertex of $G_C$ if and only if $m_{ij} \in E$ for some $i, j \in V$. Given two vertices $m, n$ in the conflict graph, $e_{mn}$ is an edge of $G_C$ with end vertices $m, n \in V_E$ if and only if $d(m_{ij}, n_{kl}) \leq K$. Also, we set the vertex weight $w_m$ in the conflict graph to be the edge weight $w_{m_{ij}}$ as specified by the Dirichlet weighting (2.1) in the origional graph. The edge weight is set as $w_{e_{mn}} = 1$. The time complexity of such conversion process is $O(|E|^2)$.

Now, by solving the Weighted Maximum Independence Set (WMIS) problem of the conflict graph, we solve the network scheduling problem of the original graph. As first proposed in [3], the WMIS problem can be formulated as the QUBO problem of

finding the minimum of the binary function

$$f(x_1, ..., x_n) = -\sum_{m\in V_E} c_m x_m + \sum_{mn\in E_C} J_{mn} x_m x_n, \quad c_m = w_m, \tag{3.1}$$

or the corresponding Ising Hamiltonian after the substitution $x_m \to (\sigma_m^z + I_{2\times 2})/2$. The binary variable $x_n = 1$ if node $n$ is in the WMIS and 0 otherwise. It is proved [3] that a sufficient condition for the ground state of the Hamiltonian to be the optimal solution to the WMIS problem is that $J_{mn} > \min(c_m, c_n)$ for $e_{mn} \in E_C$ and $c_m = w_m$, $c_n = w_n$. Thus, the solution of the QUBO problem is related to the spectrum of the corresponding Ising formulation, with the ground state energy of the Ising problem giving the solution to the network scheduling problem.

Note that the network scheduling problem has an Ising Hamiltonian with natural two-body interaction. Thus, unlike some other applications [5,6], additional reduction is not needed. Such many-body reductions would cause sizable overhead, resulting in the practical problem size that can be handled to be very small due to hardware constraints.

**4. Mapping to Physical Qubits.** After the conversion, the conflict graph is embedded in the architecture graph, so that the WMIS problem could be solved as an Ising problem. In practice, the D-Wave Chimera architecture is a specialized graph. It does not allow arbitrary coupling of two qubits. Thus, minor-embedding [3] has to be done to couple multiple physical qubits to represent one logical qubit. The minor-embedding itself is NP-hard and currently can only be done classically. There exist reasonably efficient heuristics for such process on smaller graphs [7], but it still performs poorly upon scaling.

Due to physical architectural considerations, such as limited qubit fan-out, minimizing coupler strengths, 2D chip integration etc. [8], the current hardware "Chimera" architecture is designed using $K(4,4)$ bipartite cells interconnected in a square lattice. For a problem graph to be embedded into a hardware graph, it is required that the problem graph be a subgraph of the architecture graph. In most cases, this is a very strong requirement for general problems, since the hardware graph is fixed. In the D-Wave architecture, *minor embedding* instead of *subgraph embedding* is used to allow 1-to-many vertex mapping [3]. By properly adjusting the coupling strengths of particular edges and nodes [4], more than one physical qubits can represent the same logical qubit, thus greatly increasing the range of graphs that can be minor embedded to a fixed hardware graph, at the cost of using more resources (more physical qubits).

The definition of minor embedding is as follows. Let $U$ be a fixed hardware graph. Given a problem graph $G$, the ***minor embedding*** of $G$ is defined by $\phi : G \to U$ such that (i) each vertex $v$ in $V(G)$ is mapped to a connected subtree $T_v$ of $U$; (ii) there exists a map $V(G) \times V(G) \to E(U)$ such that for each $vw \in E(G)$, there are corresponding $i_v \in V(T_v)$ and $i_w \in V(T_w)$ with $i_v i_w \in E(U)$. Minor embedding relaxes the original requirement of subgraph embedding, provided that the resources (number of physical qubits) are adequate. Crucially related to minor embedding is the concept of ***tree decomposition*** $T$ of $G$: Each vertex $i \in I$ of the tree $T$ abstracts a subset $\mathcal{V}_i$, called a "bag", of vertices of $G$ such that (i) $\cup_{i\in I}\mathcal{V}_i = V(G)$; (ii) for any $vw \in E(G)$, there is a $i \in I$ such that $v, w \in \mathcal{V}_i$; (iii) for any $v \in V$ the set $\{i \in I : v \in \mathcal{V}_i\}$ forms a connected subtree of $T$. The width of a tree

decomposition is $\max_i(|\mathcal{V}_i| - 1)$. The ***treewidth (tw)*** is the minimum width over all tree decompositions.

For several physical qubits to represent the same logical qubits, coupling strength has to be properly set as discussed in detail by Choi [3]. However, in practice, broken chains might appear as spins that represent the same logical qubit do not unanimously agree, resulting in difficulty of mapping code space solution to solution space [9]. This can usually be coped with by post processing. Figure 4.1 illustrates the overall workflow in the setup of wireless communication.

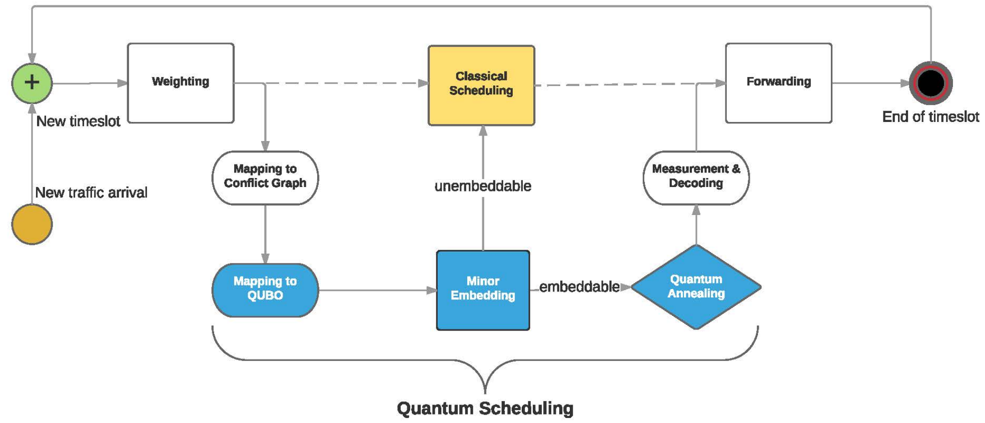


FIG. 4.1. *Workflow of quantum scheduling to replace classical scheduling, which is typically done by classical heuristics with unsatisfactory deviation of throughput from optimality. Note that not every problem is embeddable into the Chimera architecture. Thus, once a graph is unembeddable, classical scheduling heuristics can still be used. However, this is not done on our experiments, where each graph is constructed to be embeddable in order to demonstrate efficiency of D-Wave's quantum annealing.*

**5. Setting quality metrics.** To evaluate how the real world network performs under quantum scheduling, we define the **overall** quality measure of network delay, and in addition two **independent** quality measures, throughput optimality and ST99, to capture a more complete picture of quality.

Among the wireless network protocols that have been demonstrated to be throughput-optimal (e.g., Backpressure and Heat-Diffusion), network delay came out as a parameter that can be optimized subject to throughput optimality.

**5.1. Average Network Delay.** The average network delay is defined as

$$\overline{Q} = \lim_{\tau\to\infty} \sup \frac{1}{\tau} \sum_{n=0}^{\tau-1} E\left\{ \sum_{i\in V} \sum_{d\in D} q_i^{(d)}(k) \right\}, \tag{5.1}$$

with $q_i^{(d)}(k)$ denoting the $d$-class queue backlog at node $i$ at time slot $k$.

Since Poisson arrival rate is commonly assumed in wireless network studies, by Little's theorem, the expected time-averaged total queue congestion is proportional to the long-term averaged node-to-node network delay. Thus, it is sufficient to work

with average queue occupancy over all nodes in the network. The network throughput at each time slot is defined as $\sum_{ij \in E} \sum_{d \in D} f_{ij}^{(d)}(k)$, where $f_{ij}^{(d)}(k)$ is the number of $d$-packets *actually forwarded* (if interference constraints allow) along the edge $ij$ at time slot $k$. Since the exact solver for the protocol is proved to be throughput optimal, the throughput of D-Wave solution will always be less than or equal to that of the exact solver.

Next to this purely network figure of merit, we utilize another quality factor that mixes networking performance and quantum annealing performance:

**5.2. Extended Throughput Optimality.** If the returned solution is independent (no interference constraint violations), we define the quality factor of the returned solution $F_{\text{quantum}} \in [0, 1]$ as the ratio of the network throughputs that result from the quantum and the classical solver, resp. Since the classical solver is exact, the quality factor of the quantum solution has a maximum of 1. But the quantum solver might not lead to an independent solution due to errors; thus, in the case of non-independence (interference constraint violation), we define the quality factor as follows:

$$\begin{cases} F_{\text{quantum}} = \frac{\sum_{ij \in E'_s} f_{ij}}{\sum_{mn \in E'_{\text{opt}}} f_{mn}}, & \text{if quantum scheduling set solution } S \text{ has no violations,} \\ \tilde{F}_{\text{quantum}} = \frac{\sum_{ij \in E''_s} f_{ij}}{\sum_{mn \in E'_{\text{opt}}} f_{mn}}, & \text{if quantum scheduling set solution } S \text{ has violations,} \end{cases} \tag{5.2}$$

where $f$ denotes the forwarding amount as defined in section 1, $E'_S$ denotes the set of edges in the scheduling set $S$ computed by QUBO without violations and $E''_S$ the same set but with violations, and $E'_{\text{opt}}$ denotes the set of edges in the *optimal* scheduling set solved by the exact solver, without violations. If $\tilde{F}_{\text{quantum}} > 1$, there are violations that improve the throughput; if $\tilde{F}_{\text{quantum}} < 1$, there are violations but they do not even improve the throughput.

In later sections, 'throughput optimality' and 'optimality' are used interchangeably.

**5.3. ST99[OPT].** We also compare quantum annealing with simulated annealing results. Along the line of other benchmarking methods [10–12], we define a slight variant of speed measure, ST99(OPT), as the expected number of repetitions to reach at least a certain optimality level OPT with 99% certainty,

$$\begin{aligned} P_{\text{OPT}} &= \text{probability of reaching state with at least OPT optimality,} \\ ST99[\text{OPT}] &= \frac{\log(1-0.99)}{\log(1-P_{\text{OPT}})}. \end{aligned} \tag{5.3}$$

Note that this is of practical significance for time-sensitive problems like wireless network scheduling, where not enough time might be available in a time slot for the quantum annealer to reach the ground state, and quantum annealing could be of practical value if ST99[OPT] exceeds all currently available heuristics. It is also worth noting that quantum annealing gives suboptimal solutions with no additional time overhead.

Note that in the network setup, the optimality of classical heuristics heavily relies on the topology of the network and traffic rate model [2].

Table 6.1
*Search range for best parameters for simulated annealing*

| | Range |
|---|---|
| Number of sweeps | 400-10000 |
| Number of repetitions | 300-5000 |
| Initial temperature | 0.1-3 |
| Final temerature | 3-13 |
| Scheduling type | linear/exponential |

**6. SA Search Parameters.** A wide range of parameters were tested to ensure near optimal performance of the algorithm within a reasonable run time.

## REFERENCES


[1] Banirazi, R., Jonckheere, E. & Krishnamachari, B. Dirichlet's Principle on Multiclass Multihop Wireless Networks: Minimum Cost Routing Subject to Stability, ACM MSWiM, Montreal, Canada (2014, September 21-26).

[2] Wang, C., Jonckheere, E. & Banirazi, R. Interference constrained network control based on curvature, to appear, American Control Conference (2016). Available at `http://eudoxus2.usc.edu`.

[3] Choi, V. Minor-Embedding in Adiabatic Quantum Computation: I. The Parameter Setting Problem, *Quan. Info. Proc.*, 7, 193-209 (2008).

[4] Choi, V. Minor-embedding in adiabatic quantum computation: II. Minor-universal graph design, *Quan. Info. Proc.*, 10 3, 343-353 (2011).

[5] Perdomo-Ortiz, A. *et al.* Finding low-energy conformations of lattice protein models by quantum annealing, *Scientific Reports* 2, doi:10.1038/srep00571 (2012).

[6] Bian, Z. *et al.*, Experimental Determination of Ramsey Numbers, *Phys. Rev. Lett.* 111, 130505 (2013).

[7] Cai, J., Macready, W. G. & Roy, A. A practical heuristic for finding graph minors, arXiv:1406.2741.

[8] Bunyk, P.I. *et al.* Architectural considerations in the design of a superconducting quantum annealing processor, arXiv: 1401.5504.

[9] Young, K.C., Blume-Kohout, R. & Lidar, D.A.. Adiabatic quantum optimization with the wrong Hamiltonian. *Phys. Rev.* A, 88(6):062314 (2013).

[10] Boixo, S. *et al.*, Quantum annealing with more than one hundred qubits, *Nature Phys.* 10, 218 (2014).

[11] Rnnow, T. F. *et al.*, Defining and detecting quantum speedup, *Science* 345, 420 (2014).

[12] Hen, I. *et al.*, Probing for quantum speedup in spin glass problems with planted solutions, arXiv:1502.01663

[13] Tassiulas, L. and Ephremides, A. Stability Properties of Constrained Queueing Systems and Scheduling Policies for Maximum Throughput in Multihop Radio Networks, *IEEE Trans. on Auto. Con.,* vol. 37, no. 12, 1936-1948 (1992).
